# Effects of Varied Sulfamethazine Dosage and Exposure Durations on Offspring Mice

**DOI:** 10.3390/microorganisms12020381

**Published:** 2024-02-13

**Authors:** Hongchao Wang, Danting Dang, Leilei Zhu, Mingluo Pan, Jinlin Zhu, Wenwei Lu, Shourong Lu, Jianxin Zhao

**Affiliations:** 1 State Key Laboratory of Food Science and Resources, Jiangnan University, Wuxi 214122, China; hcwang@jiangnan.edu.cn (H.W.); dangdt@163.com (D.D.); 7230112006@stu.jiangnan.edu.cn (L.Z.); mingluopan@163.com (M.P.); wx_zjl@jiangnan.edu.cn (J.Z.); luwenwei@jiangnan.edu.cn (W.L.); 2 School of Food Science and Technology, Jiangnan University, Wuxi 214122, China; 3 Wuxi People’s Hospital (The Affiliated Wuxi People’s Hospital of Nanjing Medical University), Wuxi Medical Center, Nanjing Medical University, Wuxi 214023, China

**Keywords:** antibiotic, early life, sulfamethazine, gut microbiota

## Abstract

The development of antibiotics was a turning point in the history of medicine; however, their misuse and overuse have contributed to the current global epidemic of antibiotic resistance. According to epidemiological studies, early antibiotic exposure increases the risk of immunological and metabolic disorders. This study investigated the effects of exposure to different doses of sulfamethazine (SMZ) on offspring mice and compared the effects of exposure to SMZ on offspring mice in prenatal and early postnatal periods and continuous periods. Furthermore, the effects of SMZ exposure on the gut microbiota of offspring mice were analyzed using metagenome. According to the results, continuous exposure to high-dose SMZ caused weight gain in mice. IL-6, IL-17A, and IL-10 levels in the female offspring significantly increased after high-dose SMZ exposure. In addition, there was a significant gender difference in the impact of SMZ exposure on the gut microbiota of offspring: Continuous high-dose SMZ exposure significantly decreased the relative abundance of *Ligilactobacillus murinus*, *Limosilactobacillus reuteri*, *Lactobacillus johnsonii*, and *Bifidobacterium pseudolongum* (*p* < 0.05) in female offspring mice; however, these significant changes were not observed in male offspring mice.

## 1. Introduction

Gut microbiota is regarded as a “microbial organ”, and it influences the host in many aspects, including immunity, metabolism, and emotion [1,2,3]. Generally, the gut microbiota is interdependent and mutually constrained by the host to maintain a dynamic balance. However, an imbalance in the gut microbiota can cause a series of diseases such as constipation [4], diarrhea [5], inflammatory bowel disease (IBD) [6] and other intestinal diseases, obesity, diabetes, and other metabolic disorders [7]. An imbalance of the gut microbiota mostly results from medicinal intake, diet, age, and intestinal immune dysfunction, especially the long-term use of broad-spectrum antibiotics, which inhibit sensitive bacteria and cause excessive reproduction of resistant bacteria, leading to the dysfunction of the gut microbiota [8]. Antibiotics treat bacterial infections by inhibiting pathogenic bacteria; however, oral antibiotic therapies can cause a series of side effects on the gut microbiota, including the loss of composition and functional diversity, increasing host susceptibility to enteric pathogens, and promoting the spread of resistance [9,10]. A previous study indicated that the microbial diversity of patients who received broad-spectrum antibiotics decreased by 25%, the core phylogenetic microbiota decreased from 29 to 12, and the proportion of Bacteroidetes to Firmicutes increased after broad-spectrum antibiotic treatment [11].

A growing body of research indicates that pregnancy is a more intricate immune situation [12]. Therefore, the effects of antibiotic exposure during pregnancy deserve attention. According to a previous study by Hu et al. [13], prenatal antibiotic exposure affects the incidence and onset of type I diabetes in offspring. In addition, early life exposure to antibiotics leads to neurobehavioral disorders such as anxiety, sociability, and cognitive impairments in offspring mice [14]. According to a population- and register-based nested case–control study, an elevated risk of asthma in offspring is linked to the maternal use of antibiotics during pregnancy [15], and antibiotic-resistant gut bacteria can be transferred from the mother to the newborn at birth and persist for several weeks. Briefly, the use of antibiotics during pregnancy or infancy has been linked to an increased risk of obesity, inflammatory bowel disease (IBD), asthma, and other allergic/inflammatory diseases [16,17,18,19,20]. Furthermore, the effects of antibiotics on the host vary according to the sex. According to a previous study [21], prenatal low doses of penicillin in the last week of pregnancy have long-term sex-specific effects on offspring mice, with female mice exhibiting reduced anxiety-like behavior and male mice exhibiting abnormal social behavior. Additionally, prenatal exposure to penicillin can lead to different gut microbiota compositions in mice of different sexes.

Sulphonamides are a class of broad-spectrum antibiotics that are frequently used to treat animal infections and in therapeutic settings. Sulfadimidine (SMZ) is a sulfonamide antibiotic. However, sulfonamides are not easily metabolized, and approximately 50–90% of molecules enter the environment and are difficult to degrade naturally. The abuse of SMZ leads to high levels of residual sulfonamides in edible animal tissues. The overconsumption of sulphonamide-containing animal tissues leads to allergic reactions, gut microbiota dysfunction, and antibiotic resistance. According to previous studies [22,23], long-term exposure to SMZ significantly decreased the alpha diversity of marine medaka (*Oryzias melastigma*) but increased the richness and diversity of the gut microbiota of its offspring. However, previous studies have mostly focused on marine organisms, and there is limited research on the effects of SMZ exposure on the gut microbiota of rodents. Therefore, in this study, we compared the effects of prenatal and continuous exposure to different doses of SMZ in early life on serum glucose and lipids, inflammatory cytokines, immunoglobulins, and the gut microbiota of male and female offspring mice. The results showed that high-dose SMZ exposure significantly influenced the inflammatory cytokines of female offspring mice, and their gut microbiota were modified by high-dose SMZ exposure.

## 2. Materials and Methods

### 2.1. Study Design

**Figure 1:**
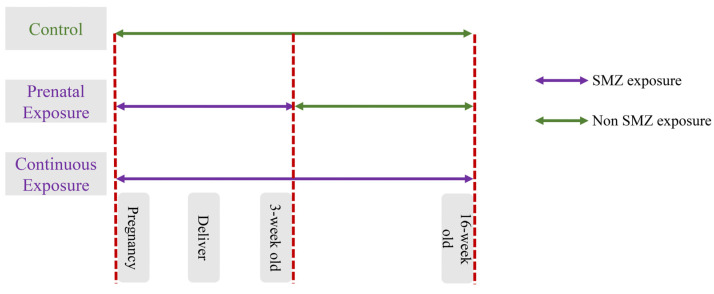
Detailed scheme of animal study design.

The Ethics Committee of Experimental Animals of Jiangnan University approved the animal trial protocol (JN. No20220330c0600101[102]). Sixty mice were purchased from Vital River Laboratory Animal Technology Co., Ltd. (Jiaxing, China) and divided randomly based on sex; they were then allowed to adapt for one week. After one-week adaption, the mice were mated in a female-to-male ratio of 2:1 from 3:30 p.m. to 5:00 p.m. It would be regarded as pregnancy for 0.5 days if a vaginal plug was detected at 7:30 a.m. the next day. If no vaginal plug was detected, mating and rest occurred for 2 to 3 days. Pregnant mice were divided into 5 groups as follows: (1) the control group, (2) the low-dose continuous exposure group (LCE), (3) the high-dose continuous exposure group (HCE), (4) the low-dose prenatal exposure group (LPE), and (5) the high-dose prenatal exposure group (HPE). The detailed scheme is shown in Figure 1. According to a previous study [16], low (30 mg/L) and high (200 mg/L) doses of SMZ were added to the water, which was changed twice a week to ensure a fresh supply of antibiotics. To study sex differences in antibiotic exposure, male and female offspring were divided at the age of 4 weeks, n = 5 per group. The feces were collected before the end of the treatment and stored at −80 °C. Offspring were sacrificed at 16 weeks of age to obtain blood, liver, and ileal samples. The partial samples of the liver and ileum were fixated in a 4% neutral paraformaldehyde solution, and the remaining tissue samples were kept at −80 °C for future analysis.

### 2.2. Biochemical Analysis

Serum samples were collected via centrifugation at 4000× *g* for 10 min. The level of glycosylated hemoglobin (Ghb), insulin (INS), immunoglobulin (IgA, IgM, IgG, and IgE), and inflammatory cytokines (IL-2, IL-6, IL-10, IL-17A, and TNF-α) in the serum were measured using corresponding enzyme-linked immunosorbent assay (ELISA) kits (Shanghai Enzyme-linked Biotechnology Co., Ltd., Shanghai, China) following the instructions. The following serum lipid indices were measured using a biochemical analyzer (AU5400, Olympus, Tokyo, Japan): total cholesterol (TC), triacylglycerol (TG), and nonesterified fatty acid (NEFA).

### 2.3. Histological Analysis

The liver and ileum tissues of the mice were fixed in a 4% neutral paraformaldehyde solution, dehydrated, and embedded in paraffin. Hematoxylin–eosin (H&E) staining was performed, followed by dehydration. Images were captured using a microscope imaging system (Pannoramic MIDI; 3DHISTECH, Budapest, Hungary).

### 2.4. DNA Extraction and Metagenomic Sequencing

For metagenomic sequencing, three mice were selected at random from each group. The FastDNA SPIN Kit for Feces (MP Biomedicals, Irvine, CA, USA) was used to extract microbial genomic DNA. Agar gel electrophoresis was performed to verify its integrity. The metagenomic sequencing of fecal samples was performed by Tianjin Novogene Co., Ltd. (Tianjin, China), and metagenomic data were determined using the MGI DNBSEQ-T7. A total of 485 Gb of raw data were generated (approximately 16.17 Gb per sample). The preprocessing of the raw data was conducted as follows: Firstly, Trimmomatic (version 0.39) was used to filter low-quality sequences; sequences with an average base quality score of less than 25 were trimmed, and sequences longer than 60 bp after filtering were kept as a quality-controlled output. Then, using Bowtie2 2.4.4, Samtools (version 1.15), and BEDTools (version 2.30.0), the filtered sequences were aligned to the human reference genome (Homo sapiens genome assembly GRCh38, hg38), therefore eliminating host-origin genes from the samples. After filtering out low-quality data and host contamination, 277 Gb of high-quality clean data were generated. MetaPhlAn3 and HUMAnN3 were used for the species and functional annotation of high-quality data, and then the results were standardized to relative abundance. Alpha diversity was shown using the richness and Shannon indices. Principal coordinate analysis (PCoA) plots and nonmetric multidimensional scaling (NMDS) were used to indicate and display beta diversity. LEfSe (linear discriminant analysis (LDA) effect size) was performed online (https://huttenhower.sph.harvard.edu/galaxy/ accessed on 30 May 2023). Raw data are available on NCBI (BioProject Accession Number: PRJNA1066086).

### 2.5. Statistical Analysis

Significant analysis was performed using a one-way ANOVA test by GraphPad Prism 8 software, and data are shown using mean ± SD. The correlation index was calculated using Spearman’s correlation method with OmicShare tools, a free online platform for data analysis (http://www.omicshare.com/tools accessed on 30 May 2023). Differences were considered statistically significant at *p* < 0.05.

## 3. Results

### 3.1. Effects of SMZ Exposure on Body Weight of Offspring Mice

**Figure 2:**
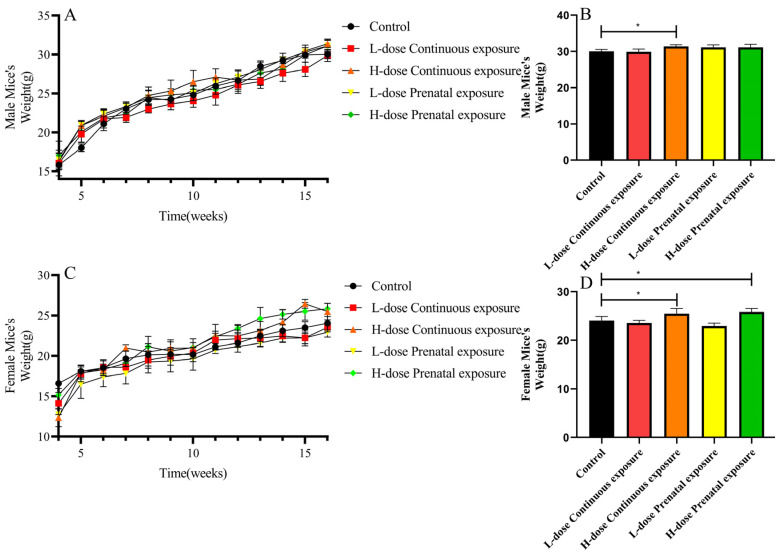
Effects of SMZ exposure on body weight of offspring mice: (**A**) body weight of female mice; (**B**) body weight of male mice; (**C**) body weight in 16-week-old male mice; (**D**) body weight in 16-week-old female mice.* indicates a significant difference between the two groups (*p* < 0.05).

The offspring mice of all five groups steadily increased in body weight (Figure 2A,C). However, in the 16th week, the body weight of the male mice of the HCE group was significantly higher than the control group (Figure 2B, 31.38 ± 0.48 g vs. 30.02 ± 0.55 g, *p* < 0.05). Moreover, the 16-week-old female offspring mice of the HCE and HPE groups were significantly heavier than the offspring mice of the control group (Figure 2D, 25.50 ± 1.03 g vs. 24.07 ± 0.85 g and 25.84 ± 0.70 g vs. 24.07 ± 0.85 g, respectively, *p* < 0.05).

### 3.2. Effects of SMZ Exposure on Blood Glucose of Offspring Mice

**Figure 3:**
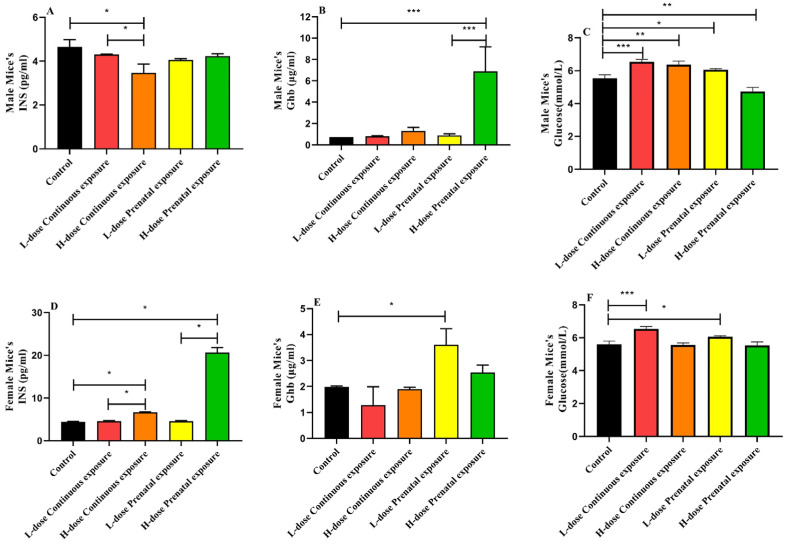
Effects of SMZ exposure on blood glucose of offspring mice: (**A**) serum INS of male mice; (**B**) serum Ghb of male mice; (**C**) serum glucose of male mice; (**D**) serum INS of female mice; (**E**) serum Ghb of female mice; (**F**) serum glucose of female mice; * indicates a significant difference between the two groups (*p* < 0.05); ** indicates *p* < 0.01; *** indicates *p* < 0.001.

In male offspring mice, the INS concentration of the mice in the HCE group was significantly lower than that of the control group (Figure 3A, *p* < 0.05), and there was a significant difference in INS levels between the HCE and LCE groups (*p* < 0.05). The Ghb levels in the HPE group were significantly higher than those in the control and LPE groups (Figure 3B, *p* < 0.05). The effects of SMZ exposure on the blood glucose of male offspring mice are shown in Figure 3C. Compared with the control group, the blood glucose levels of mice in the LCE, HCE, and LPE groups were significantly increased, while those in the HPE group were significantly decreased.

For female offspring, the INS levels of the HPE and HCE groups were significantly higher than those of the control group (Figure 3D, *p* < 0.05), and there were significant differences between the HCE and LCE groups and between the HPE and LPE groups (*p* < 0.05). In addition, compared with the control group, Ghb levels significantly increased after low-dose prenatal exposure (Figure 3E, *p* < 0.05). In addition, the blood glucose levels of female mice in the LCE and LPE groups were significantly higher than those in the control group (Figure 3F, *p* < 0.05).

### 3.3. Effects of SMZ Exposure on Serum Lipids of Offspring Mice

**Figure 4:**
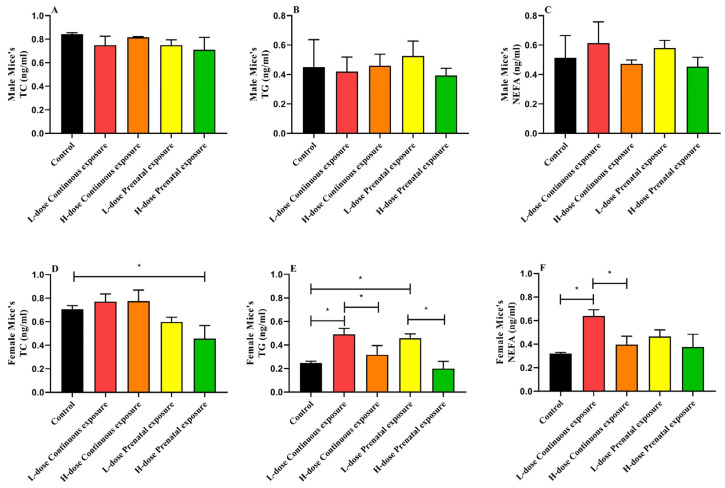
Effects of SMZ exposure on serum lipids of offspring mice: (**A**) serum TC of male mice; (**B**) serum TG of male mice; (**C**) serum NEFA of male mice; (**D**) serum TC of female mice; (**E**) serum TG of female mice; (**F**) serum NEFA of female mice; * indicates a significant difference between the two groups (*p* < 0.05).

Compared with the control group, there were no significant differences in the TC, TG, and NEFA levels in male offspring after SMZ exposure (Figure 4A–C). However, in female offspring, high-dose prenatal exposure significantly decreased the level of serum TC compared to that in the control group (Figure 4D, *p* < 0.05). In addition, the serum TG levels in the LCE and LPE groups were significantly higher than those in the control group (Figure 4E, *p* < 0.05), and there were significant differences between the HCE and LCE groups and between the HPE and LPE groups (*p* < 0.05). Serum NEFA levels in the LCE group were significantly higher than those in the control and HCE groups (Figure 4F, *p* < 0.05).

### 3.4. Effects of SMZ Exposure on Histopathology of Offspring Mice

**Figure 5:**
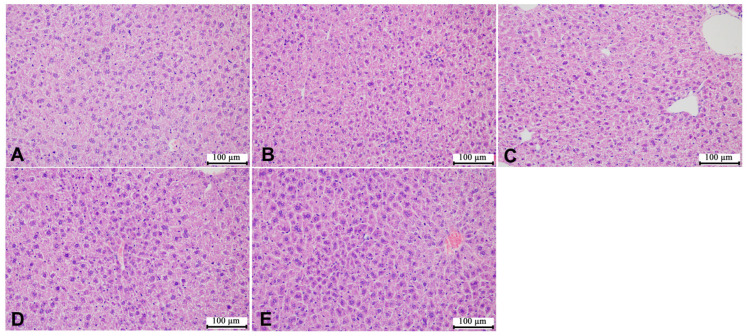
Representative images of liver H&E staining of male offspring mice: (**A**) the control group; (**B**) the LCE group; (**C**) the HCE group; (**D**) the LPE group; (**E**) the HPE group. Magnification: ×200.

**Figure 6:**
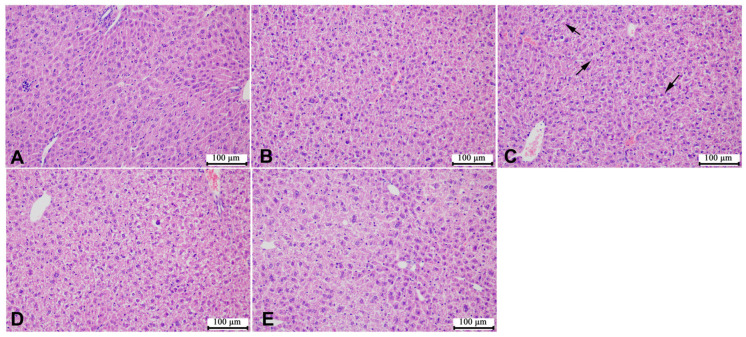
Representative images of liver H&E staining of female offspring mice: (**A**) the control group; (**B**) the LCE group; (**C**) the HCE group, black arrows point the gaps in the cell arrangement; (**D**) the LPE group; (**E**) the HPE group. Magnification: ×200.

**Figure 7:**
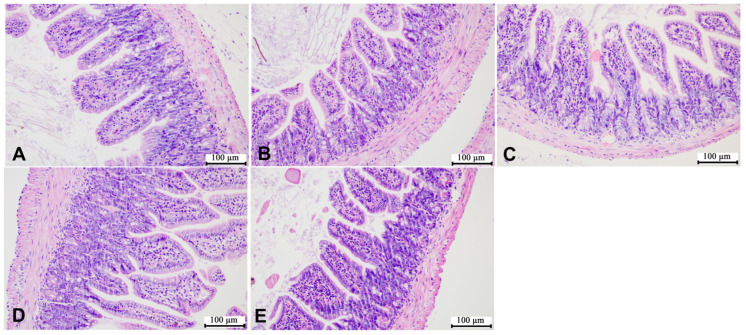
Representative images of ileum H&E staining of male offspring mice: (**A**) the control group; (**B**) the LCE group; (**C**) the HCE group; (**D**) the LPE group; (**E**) the HPE group. Magnification: ×200.

**Figure 8:**
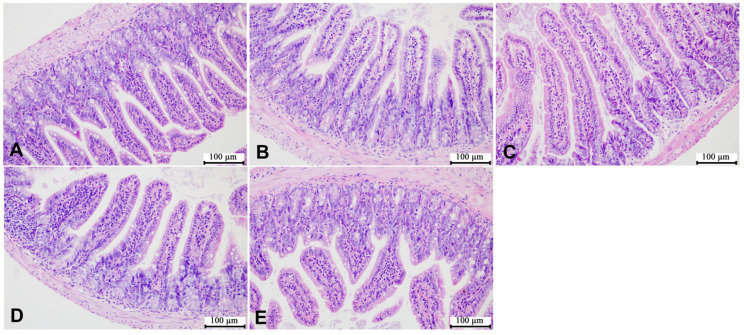
Representative images of ileum H&E staining of female offspring mice: (**A**) the control group; (**B**) the LCE group; (**C**) the HCE group; (**D**) the LPE group; (**E**) the HPE group. Magnification: ×200.

According to the representative images of liver H&E staining shown in Figure 5 and Figure 6, the liver cells of the mice in the control group were of the same size, with complete and tightly arranged structures, and there was no significant difference between the male mice in each group and the mice in the control group. However, the female mice in the HCE group showed few gaps in the cell arrangement. In addition, as shown in Figure 7 and Figure 8, SMZ exposure did not damage the ileum tissue of the mice, since the ileal mucosa of each group of male and female mice was intact, with long villi arranged neatly, and without any inflammation.

### 3.5. Effects of SMZ Exposure on Immunoglobulin of Offspring Mice

**Figure 9:**
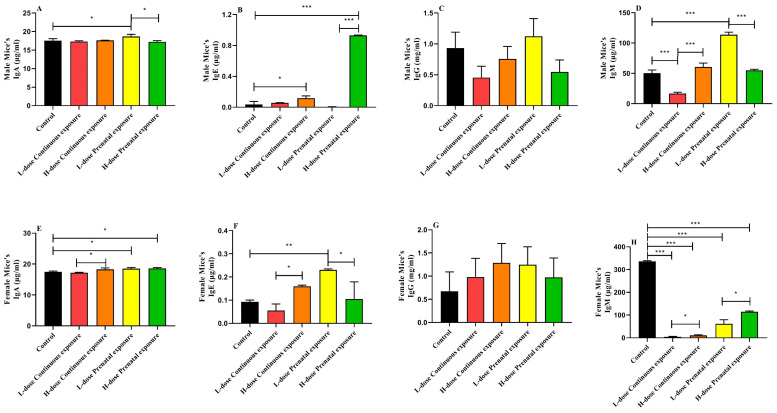
Effects of SMZ exposure on immunoglobulin of offspring mice: (**A**) serum IgA of male mice; (**B**) serum IgE of male mice; (**C**) serum IgG of male mice; (**D**) serum IgM of male mice; (**E**) serum IgA of female mice; (**F**) serum IgE of female mice; (**G**) serum IgG of female mice; (**H**) serum IgM of female mice; * indicates a significant difference between the two groups (*p* < 0.05); ** indicates *p* < 0.01; *** indicates *p* < 0.001.

For male offspring, the serum IgA level of mice in the LPE group was significantly higher than that of the mice in the control and HPE groups (Figure 9A, *p* < 0.05). High-dose prenatal exposure to SMZ significantly increased serum IgE levels compared with the control and low-dose prenatal exposure groups (Figure 9B, *p* < 0.05). In addition, exposure to SMZ did not significantly influence the serum IgG level (Figure 9C). However, the IgM level of the mice in the LCE group was significantly lower than that of the control and HCE groups (Figure 9D, *p* < 0.05), and the IgM level of the LPE group was significantly higher than that of the control and HPE groups (Figure 9D, *p* < 0.05).

In contrast to the male mice, for female offspring, both low- and high-dose prenatal exposure to SMZ significantly increased the IgA level (Figure 9E, *p* < 0.05), and the IgA level of the HCE group was significantly higher than that of the LCE group (*p* < 0.05). In addition, the IgE levels of the female offspring in the LPE group were significantly higher than those in the control group (Figure 9F, *p* < 0.05). Similar to the male mice, exposure to SMZ did not significantly influence the serum IgG levels in the female mice (Figure 9G). Furthermore, compared with the control group, SMZ exposure significantly decreased the level of IgM in all four groups (Figure 9H, *p* < 0.05).

### 3.6. Effects of SMZ Exposure on Inflammatory Cytokines of Offspring Mice

**Figure 10:**
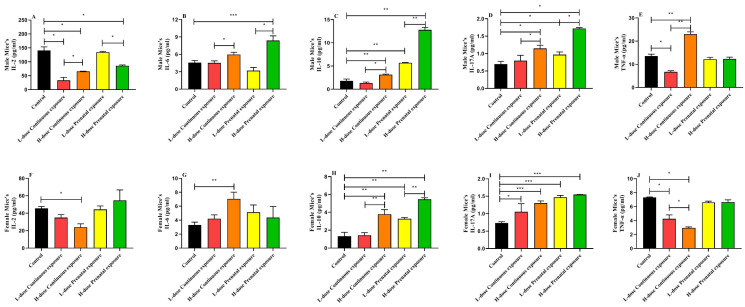
Effects of SMZ exposure on inflammatory cytokines of offspring mice: (**A**) serum IL-2 of male mice; (**B**) serum IL-6 of male mice; (**C**) serum IL-10 of male mice; (**D**) serum IL-17A of male mice; (**E**) serum TNF-α of male mice; (**F**) serum IL-2 of female mice; (**G**) serum IL-6 of female mice; (**H**) serum IL-10 of female mice; (**I**) serum IL-17A of female mice; (**J**) serum TNF-α of female mice; * indicates a significant difference between the two groups (*p* < 0.05); ** indicates *p* < 0.01; *** indicates *p* < 0.001.

In the male mice, the serum IL-2 levels in the LCE, HCE, and HPE groups were significantly lower than those in the control group (Figure 10A, *p* < 0.05). High-dose exposure to SMZ significantly increased the level of serum IL-6 (Figure 10B, *p* < 0.05), and high-dose continuous or prenatal exposure to SMZ significantly increased IL-6 levels compared to low-dose continuous or prenatal exposure (*p* < 0.05). Except for the mice in the LCE group, IL-10 and IL-17A levels in the other three groups were significantly higher than those in the control group (Figure 10C,D, *p* < 0.05). Moreover, compared with the control group, continuous low-dose exposure to the SMZ significantly decreased the level of TNF-α, whereas continuous high-dose exposure to the SMZ significantly increased it (Figure 10E, *p* < 0.05).

In the female mice, compared to the control group, the level of IL-2 was significantly decreased, and the level of IL-6 was significantly increased after continuous high-dose exposure (Figure 10F,G, *p* < 0.05). Similar to the male mice, except for the mice in the LCE group, IL-10 levels in the other three groups were significantly higher than those in the control group (Figure 10H, *p* < 0.05). In addition, IL-17A levels in the four SMZ exposure groups were significantly higher than those in the control group (Figure 10I, *p* < 0.05). In addition, the serum TNF-α level was significantly decreased after continuous low- or high-dose exposure to SMZ, and the TNF-α level in the HCE group was significantly lower than that of the LCE group (Figure 10J, *p* < 0.05).

### 3.7. Effects of SMZ Exposure on Diversity of Gut Microbiota of Offspring Mice

**Figure 11:**
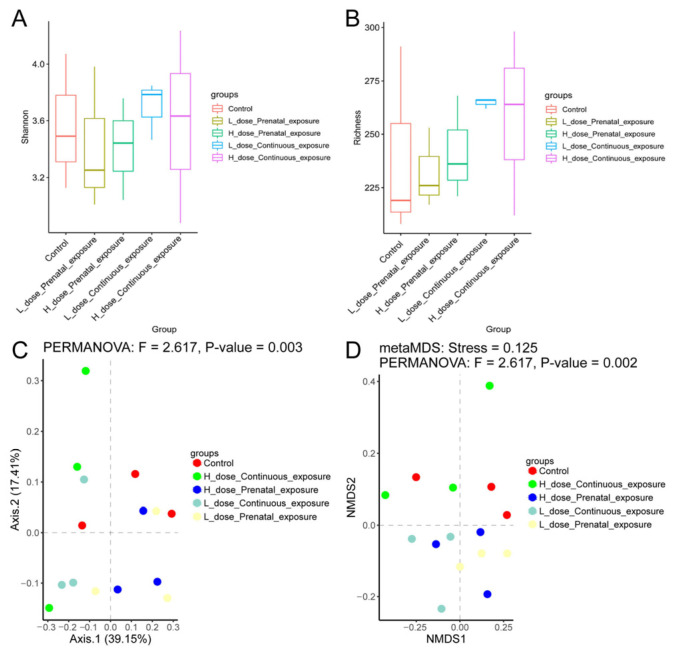
Effects of SMZ exposure on diversity of gut microbiota of male offspring mice: (**A**) Shannon index; (**B**) richness index; (**C**) PCoA of gut microbiota of male offspring mice; (**D**) NMDS of gut microbiota of male offspring mice.

According to the results of alpha diversity in Figure 11, SMZ exposure did not influence the Shannon and richness indices of the gut microbiota of male offspring mice (Figure 11A,B). Furthermore, beta diversity was calculated using Bray–Curtis-based PCoA and NMDS. The results showed that each group was clustered and differed from each other significantly according to the PERMANOVA analysis (*p* = 0.003); moreover, the stress value of NMDS analysis was 0.125, indicating that the results of NMDS analysis had good explanatory significance (Figure 11C,D).

**Figure 12:**
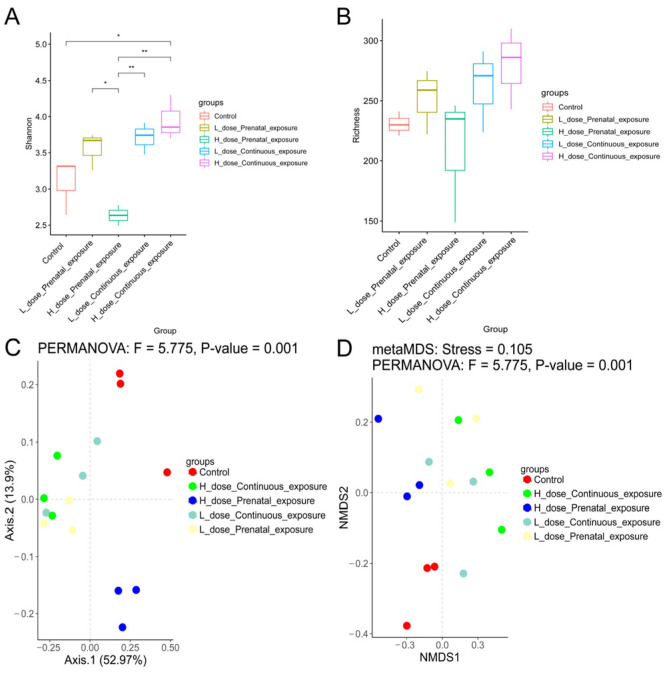
Effects of SMZ exposure on diversity of gut microbiota of female offspring mice: (**A**) Shannon index; (**B**) richness index; (**C**) PCoA of gut microbiota of female offspring mice; (**D**) NMDS of gut microbiota of female offspring mice; * indicates a significant difference between the two groups (*p* < 0.05); ** indicates *p* < 0.01.

Different from the male mice, compared with the female mice in the control group, continuous high-dose exposure to SMZ significantly increased the Shannon index of the female mice (*p* < 0.05). Additionally, the Shannon index of the LPE group was significantly higher than that of the HPE group (Figure 12A, *p* < 0.05). However, there was no significant difference in the richness indices among the five groups (Figure 12B). In addition, beta diversity analysis indicated that each group was clustered and differed from each other significantly (Figure 12C,D, *p* = 0.001).

### 3.8. Effects of SMZ Exposure on the Composition of Gut Microbiota of Offspring Mice

**Figure 13:**
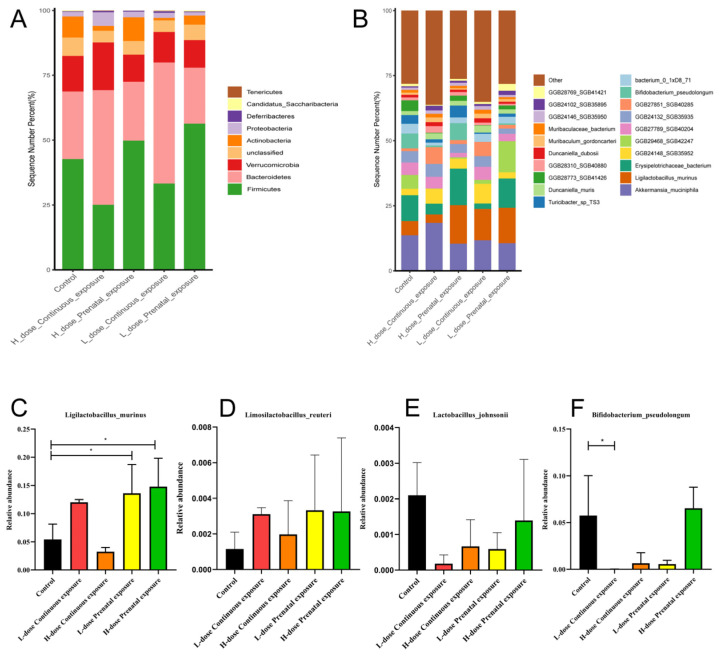
Effects of SMZ exposure on composition of gut microbiota of male offspring mice: (**A**) microbial distribution at the phylum level; (**B**) microbial distribution at the genus level; (**C**) relative abundance of *Ligilactobacillus murinus*; (**D**) relative abundance of *Limosilactobacillus reuteri*; (**E**) relative abundance of Lactobacillus johnsonii; (**F**) relative abundance of *Bifidobacterium pseudolongum*; * indicates a significant difference between the two groups (*p* < 0.05).

The composition of the gut microbiota of the male mice in each group is shown in Figure 13. Compared to the control group, the relative abundance of Firmicutes gradually decreased, whereas the relative abundance of Bacteroidetes gradually increased as the dose increased in the LCE and HCE groups, and the relative abundance of Actinobacteria decreased in the LCE and HCE groups (Figure 13A). In addition, the relative abundances of *Erysipelotrichaceae bacterium* and *Bifidobacterium pseudolongum* decreased, and the relative abundances of *Muribaculum gordoncarter* and *Muribaculaceae bacterium* increased in the LCE and HCE groups, without significance (Figure 13B). Furthermore, compared with the control group, the relative abundance of *Ligilactobacillus murinus* was significantly increased in the LPE and HPE group (Figure 13C, *p* < 0.05), and the relative abundance of *Bifidobacterium pseudolongum* significantly decreased in the LCE group (Figure 13F, *p* < 0.05). Moreover, the relative abundance of *Limosilactobacillus reuteri* and *Lactobacillus johnsonii* changed without significance (Figure 13D,E).

**Figure 14:**
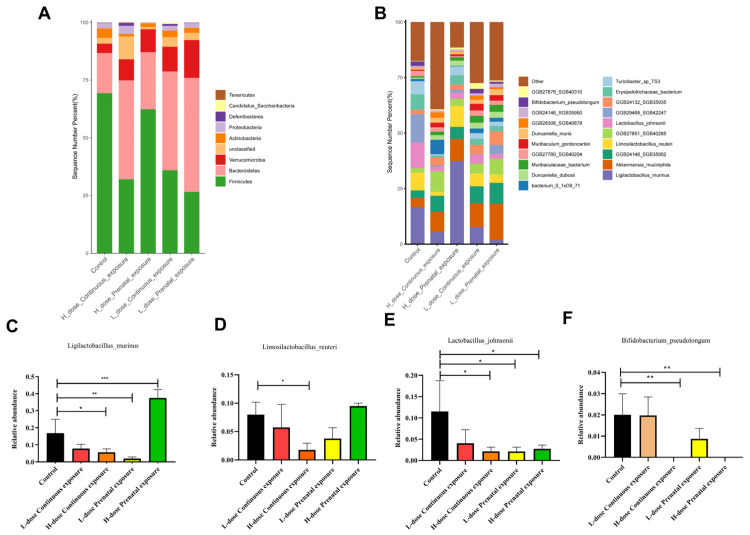
Effects of SMZ exposure on composition of gut microbiota of female offspring mice: (**A**) microbial distribution at the phylum level; (**B**) microbial distribution at the genus level; (**C**) relative abundance of *Ligilactobacillus murinus*; (**D**) relative abundance of *Limosilactobacillus reuteri*; (**E**) relative abundance of Lactobacillus johnsonii; (**F**) relative abundance of *Bifidobacterium pseudolongum*; * indicates a significant difference between the two groups (*p* < 0.05); ** indicates *p* < 0.01; *** indicates *p* < 0.001.

For female offspring, compared with the control group, the relative abundance of Firmicutes gradually decreased, whereas the relative abundance of Bacteroidetes gradually increased as the dose increased in the mice of the LCE and HCE groups, and the relative abundance of Actinobacteria increased in all four groups (Figure 14A). In addition, compared to the control group, the relative abundances of *Ligilactobacillus murinus*, *Limosilactobacillus reuteri*, *Lactobacillus johnsonii*, and *Bifidobacterium pseudolongum* decreased, whereas the relative abundances of *Muribaculum gordoncarter* and *Muribaculaceae bacterium* increased in the LCE and HCE groups (Figure 14B). Furthermore, compared with the control group, the relative abundance of *Ligilactobacillus murinus* significantly decreased in the HCE and LPE groups but significantly increased in the HPE group (Figure 14C, *p* < 0.05). The relative abundance of *Limosilactobacillus reuteri* significantly decreased in the HCE group (Figure 14D, *p* < 0.05); the relative abundance of *Lactobacillus johnsonii* significantly decreased in the HCE, LPE, and HPE groups (Figure 14E, *p* < 0.05); and the relative abundance of *Bifidobacterium pseudolongum* significantly decreased in the HCE and HPE groups (Figure 14F, *p* < 0.05).

### 3.9. Effects of SMZ Exposure on Characteristic Microbiota of Offspring Mice

**Figure 15:**
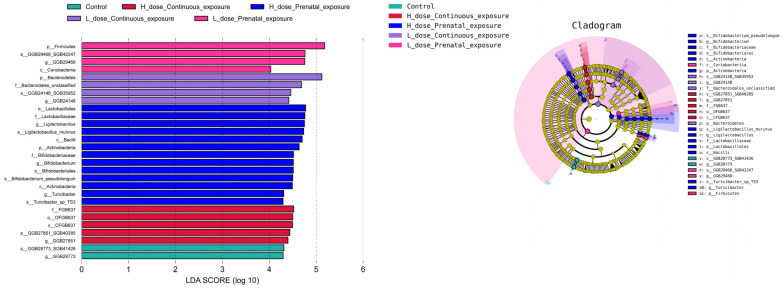
Gut microbiota biomarkers based on the LEfSe analysis of male mice.

According to the LEfSe analysis of the gut microbiota of the male offspring mice (Figure 15), the characteristic microbiota biomarkers of the HPE group were s_*Ligilactobacillus murinus*, s_*Bifidobacterium*_*pseudolongum* and s_*Turiobacter*_sp_TS3, whereas the characteristic microbiota of the LPE group was s_GGB29468_SGB42247. The characteristic microbiota biomarkers of the LCE and HCE groups were s_GGB24148_SGB35952 and s_GGB27851_SGB40285, respectively.

**Figure 16:**
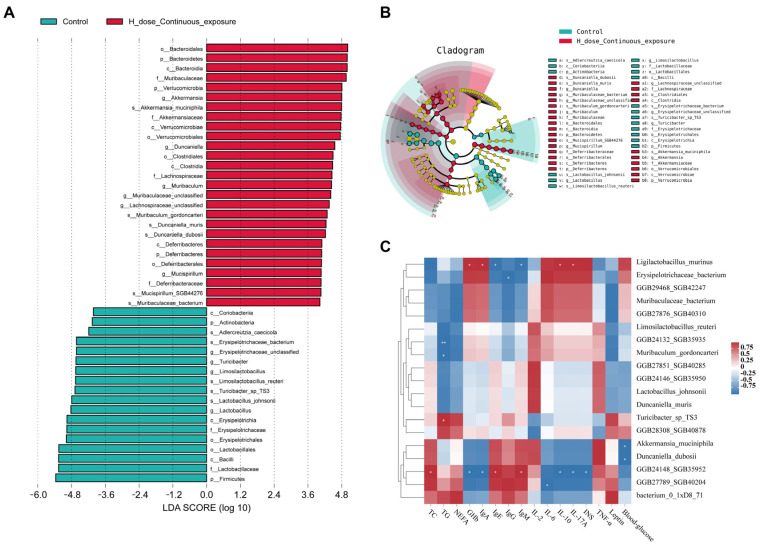
Gut microbiota biomarkers based on LEfSe analysis of female mice: (**A**) histogram of LEfSe analysis of female mice; (**B**) cladogram of LEfSe analysis of female mice; (**C**) heatmap of the relevance analysis between TOP 20 genus and biochemical indicators of the HCE group; * indicates *p* < 0.05; ** indicates *p* < 0.01.

In female offspring continuously exposed to high doses, the characteristic microbiota biomarkers were Mucispirillum SGB44276, *Duncaniella dubosii*, *Duncaniella muris*, *Akkermansia muciniphila*, *Muribaculaceae bacterium*, and *Muribaculum gordoncarteri* (Figure 16A,B). Furthermore, according to the relevance analysis between the TOP 20 genera and the biochemical indicators of the HCE group, the relative abundance of *Ligilactobacillus murinus* was significantly correlated with the levels of Ghb, IgA, IL-10, IL-17A, IgE, and IgM (Figure 16C, *p* < 0.05).

### 3.10. Effects of SMZ Exposure on Metabolic Pathways of Gut Microbiota of Offspring Mice

**Figure 17:**
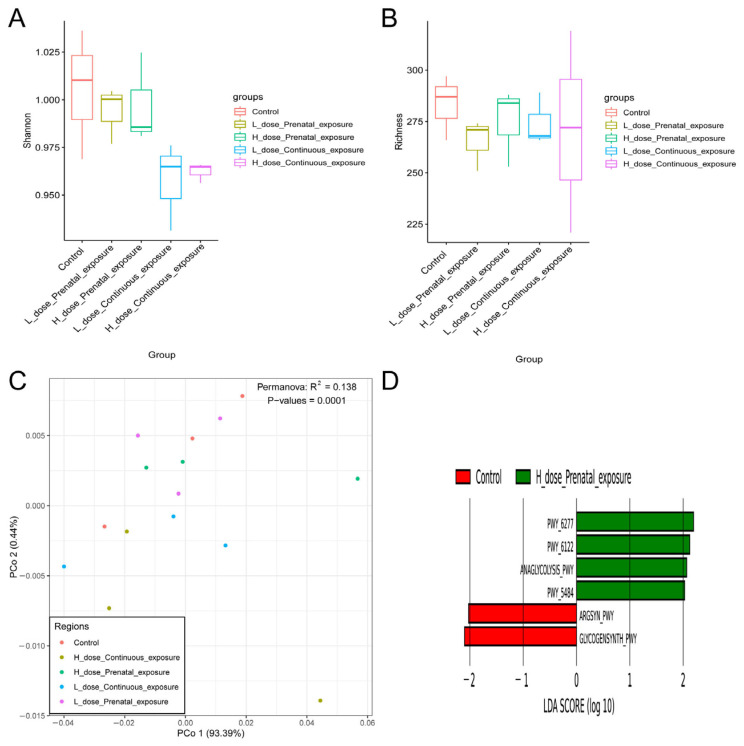
Effects of SMZ exposure on metabolic pathways of gut microbiota of male offspring mice: (**A**) Shannon index; (**B**) richness index; (**C**) PCoA of metabolic pathways of male offspring mice; (**D**) LEfSe analysis of metabolic pathways.

For the male mice, there were no significant differences in the Shannon and richness indices between the groups (Figure 17A,B). In addition, PCoA indicated that each group was clustered and significantly different from the others (Figure 17C, *p* = 0.0001). Furthermore, according to LEfSe analysis, there were four metabolic pathways significantly different from other groups in the HPE group, namely PWY-6277: superpathway of 5-aminoimidazole ribonucleotide biosynthesis; 5-aminoimidazole ribonucleotide biosynthesis II; ANAGLYCOLYSIS-PWY: glycolysis III (from glucose); and PWY-5484: glycolysis II (from fructose 6-phosphate).

**Figure 18:**
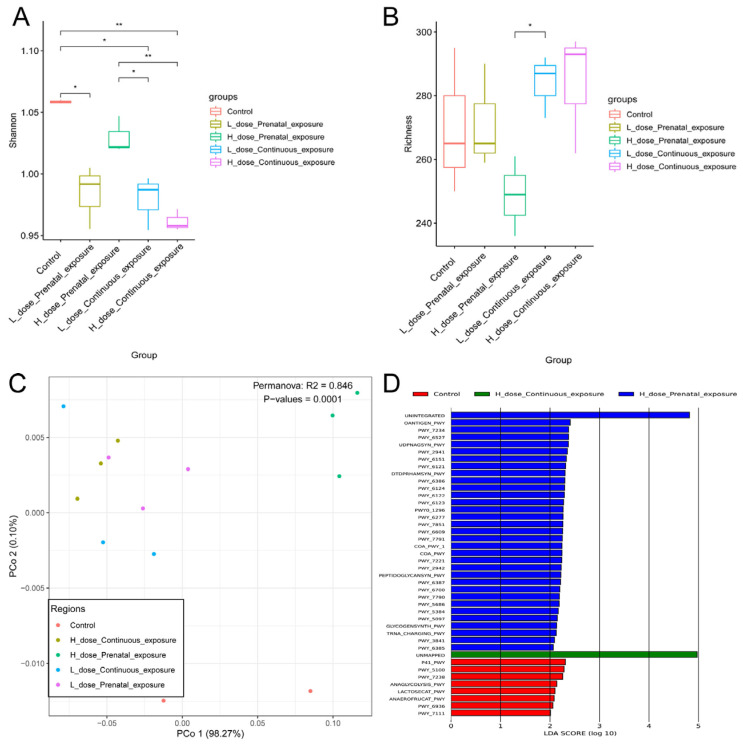
Effects of SMZ exposure on metabolic pathways of gut microbiota of female offspring mice: (**A**) Shannon index; (**B**) richness index; (**C**) PCoA of metabolic pathways of female offspring mice; (**D**) LEfSe analysis of metabolic pathways; * indicates a significant difference between the two groups (*p* < 0.05); ** indicates *p* < 0.01.

For the female mice, the Shannon indices of the LPE, LCE, and HCE groups were significantly lower than those of the control group (Figure 18A, *p* < 0.05), and there was no significant difference between the four groups and the control group in the richness index (Figure 18B). In addition, PCoA indicated that each group was clustered and significantly different from the others (Figure 18C, *p* = 0.001). Furthermore, there were characteristic metabolic pathways in the HPE group, namely OANTIGEN-PWY: O-antigen building blocks biosynthesis; PWY-7234: inosine-5′-phosphate biosynthesis III; and UDPNAGSYN-PWY: UDP-N-acetyl-D-glucosamine biosynthesis I.

## 4. Discussion

This study focused on the effects of different doses and periods of SMZ exposure on the biochemical indicators and gut microbiota of offspring mice. The development of antibiotics is widely regarded as one of the greatest medical advancements of the 20th century, but their excessive use and abuse by humans, veterinarians, and animal husbandry have led to the current global crisis of antibiotic resistance, which reduces our ability to prevent and treat bacterial infections [24,25]. It was reported that broad-spectrum antibiotics caused changes in the composition and diversity of the gut microbiota [26,27]. In this study, the alfa diversity and beta diversity were changed after SMZ treatment. Furthermore, in agreement with other studies, we found significant sex differences in the effect of SMZ exposure on the gut microbiota of offspring. Yurkovetskiy et.al [28] indicated that gender differences in the composition of gut microbiota resulted from the effect of sexual hormones on the production of bile acids, further affecting the gut microbiota [29]. In the female offspring mice, the Shannon index of the HPE group significantly decreased, while that of the HCE group significantly increased compared to that of the control group. The LEfSe analysis of metabolic pathways of the gut microbiota of female offspring mice indicated that the levels of OANTIGEN-PWY:O-antigen-building block biosynthesis and UDPNAGSYN-PWY: UDP-N-acetyl-D-glucosamine biosynthesis I in the HPE group were significantly higher than those in the other groups. These two metabolic pathways are related to the synthesis of lipopolysaccharides and the composition of bacterial cell walls. Therefore, SMZ treatment may reduce the diversity of the gut microbiota of female offspring mice by disrupting the formation of bacterial cell walls. Furthermore, in female offspring, the relative abundances of *Lactobacillus* and *Bifidobacterium* were significantly decreased after continuous high-dose exposure to SMZ, specifically *Ligilactobacillus murinus*, *Limosilactobacillus reuteri*, *Lactobacillus johnsonii*, and *Bifidobacterium pseudolongum*. *Ligilactobacillus murinus* is widely distributed in the intestines of rats, mice, dogs, and humans [30,31]. Previous studies have indicated that *Ligilactobacillus murinus* decreases the incidence of late-onset sepsis in premature infants [32]. In addition, *Lactobacillus murinus* HU-1 rescued behavioral deficits and activated brain region-specific microglia in the offspring of mice, resulting from maternal microbiome dysbiosis [33]. In addition, *Lactobacillus murinus* promoted the release of IL-10 from macrophages through TLR2 to prevent ischemia/reperfusion injury in mice [34]. *Lactobacillus johnsonii* is widely distributed in the intestines of humans [35]. A previous study indicated that *Lactobacillus johnsonii* could relieve experimental colitis by activating native macrophages and releasing IL-10 [36]. Furthermore, *Limosilactobacillus reuteri* lowers cholesterol levels by increasing bile acid degradation [37]. In this study, the relative abundance of *Limosilactobacillus reuteri* in the gut microbiota of the female mice significantly decreased in the HCE group, and the levels of serum TG and NEFA significantly increased, indicating a relationship between the relative abundance of *Limosilactobacillus reuteri* and serum lipid levels after SMZ treatment. *Bifidobacterium pseudolongum* is a highly abundant *Bifidobacterium* in the intestine, with an average relative abundance of over 10% in various hosts [38]. A previous study indicated that *Bifidobacterium pseudolongum* significantly decreased body weight, TG levels, and energy intake in mice fed a high-fat diet by modulating the gut microbiota [39]. In this study, the relative abundance of *Bifidobacterium pseudolongum* decreased in high-dose treatment groups, and the body weight of mice was significantly higher than that in the control group; thus, the underlying mechanism deserves further exploration.

Changes in the composition of gut microbiota result in changes in metabolism and immunity. There is increasing concern that antibiotic exposure may have long-term consequences. Cho et al. [16] reported that the fat accumulation rate in male and female mice significantly increased after subtherapeutic antibiotic treatment. A cohort study showed that if mothers received broad-spectrum antibiotics in the middle of pregnancy, their children were significantly overweight by the age of 7 [40]. The body weight of offspring mice that maternal mice prenatal and offspring mice continuous exposure to different dose of SMZ for 4–16 week was measured in this study. Consistent with other studies, the body weight of 16-week-old female offspring in the HPE and HCE groups was significantly higher than that of the control group, and the body weight of 16-week-old male offspring continuously exposed to SMZ was significantly increased. Low-dose SMZ exposure did not have a significant impact on the weight of the offspring in this study. Dose-related effects on body weight may result from different effects on the gut microbiota, as previously mentioned. These results are consistent with a previous study [41] about exposure to penicillin G (Pen G) and erythromycin (Ery), which indicated that the body weight of mice treated with high levels of Ery or Pen G significantly increased, indicating that, regarding the use of antibiotics, more attention should be given to the dosage and course of treatment.

The impact of the accidental ingestion of antibiotics on pregnant women was investigated in a previous study [42], which indicated that glucose metabolism and insulin levels of pregnant women exposed to antibiotics were higher than those of nonexposed pregnant women and that antibiotic exposure resulted in changes in the gut microbiota. Additionally, according to reports [43], fluoroquinolones cause hypoglycemia by stimulating the release of insulin from pancreatic beta cells by blocking K(ATP) channels and opening L-type voltage-dependent Ca(2+) channels. In addition, some fluoroquinolone antibiotics, such as tefloxacin and gatifloxacin, are recommended to be withdrawn from the US market because they cause a significant decrease in blood glucose [44]. Most studies have reported that the antibiotics related to blood glucose are fluoroquinolones, and there are few reports on the effects of sulfonamide antibiotics on blood glucose levels. In this study, the insulin level of the male offspring mice in the HCE group was significantly lower than that of the control group, the blood glucose level of the HCE group was significantly higher, and the levels of the two glycolytic pathways in the HCE group were significantly higher than those in the other groups (ANAGLYCOLYSIS-PWY: glycolysis III (from glucose) and PWY-5484: glycolysis II (from fructose 6-phosphate)). Conversely, the insulin levels in the female mice in the HCE group were significantly increased, their blood glucose levels were decreased, and the dosage of SMZ was positively correlated with the insulin concentration. Surprisingly, the insulin level of offspring mice after prenatal exposure to SMZ was nearly five times higher than that of the control group, which may result from the insulin resistance caused by sulphonamide antibiotics; however, due to the lack of relevant reports, the mechanisms of changes in insulin and blood glucose levels caused by sulphonamide antibiotics should be further investigated.

Antibiotic-induced microbiota disturbances can impair intestinal homeostasis and the integrity of intestinal defenses, which protect against invasive pathogens and inflammatory bowel disease [45]. The levels of IL-6, IL-10, IL-17, and TNF-α were related to intestinal barrier function and intestinal inflammation [46,47,48]. In this study, the levels of TNF-α, IL-10, and IL-17A of the male mice in the HCE group were significantly higher than those of the control group, and for the female mice, after continuous high-dose exposure to SMZ, the levels of IL-6, IL-10, and IL-17A significantly increased, while the level of TNF-α significantly decreased compared with the control group. However, the pathological results showed no significant inflammation in either group. According to a previous study [49], after one week of antibiotic treatment, three-week-old mice were more susceptible to peanut allergy, with increased serum IgE levels. Russell et al. exposed mice to vancomycin starting from pregnancy and lasting for several weeks after birth, resulting in the exacerbation of allergic inflammation in the offspring, including an increase in the number of pulmonary eosinophils and elevated serum IgE concentrations [50]. Similar to other studies, continuous high-dose exposure to SMZ significantly increased IgE levels in male mice. 

## 5. Conclusions

The body weights of the offspring increased as a result of continuous exposure to high doses of SMZ. After high-dose SMZ exposure, the levels of IL-6, IL-17A, and IL-10 in the female progeny mice significantly increased. Additionally, the effects of SMZ exposure on the gut microbiota of offspring mice varied significantly depending on the gender. SMZ exposure significantly decreased the relative abundance of *Ligilactobacillus murinus*, *Limosilactobacillus reuteri*, *Lactobacillus johnsonii*, and *Bifidobacterium pseudolongum* but significantly increased the relative abundance of *Duncaniella dubosii*, *Duncaniella muris*, *Akkermansia muciniphila*, and *Muribaculaceae bacterium* in female offspring mice. In summary, these findings suggest a potential link between SMZ exposure, changes in cytokine levels, and alterations in gut microbiota composition, emphasizing the relevance of the study to understanding immunological and metabolic disorders.

## Data Availability

All data are provided in full in this paper.
